# Efficient Delivery of Investigational Antibacterial Agents via Sustainable Clinical Trial Networks

**DOI:** 10.1093/cid/ciw244

**Published:** 2016-08-01

**Authors:** Anthony McDonnell, John H. Rex, Herman Goossens, Marc Bonten, Vance G. Fowler, Aaron Dane

**Affiliations:** 1 UK Review on Antimicrobial Resistance, London; 2 AstraZeneca Pharmaceuticals, Waltham, Massachusetts; 3 University of Texas Medical School–Houston; 4 Laboratory of Medical Microbiology, VAXINFECTIO, University Hospital of Antwerp, Belgium; 5 Department of Medical Microbiology, Julius Center for Health Sciences and Primary Care, University Medical Center Utrecht, The Netherlands; 6 Division of Infectious Diseases and Duke Clinical Research Institute, Duke University Medical Center, Durham, North Carolina;; 7 AstraZeneca Pharmaceuticals, Alderley Park, United Kingdom

**Keywords:** antibiotic development, antimicrobial resistance, trial networks

## Abstract

The economics of antibiotics can be improved by infectious diseases–specific clinical trial networks. While developers would still need to implement an independent phase 1 program as well as studies focused on highly resistant pathogens, standardized procedures in a network focused on usual drug resistance phenotype isolates would permit sharing of controls and would predictably generate high-quality pivotal data for product registration while creating cost and time savings in the range of 30%–40%. This would reduce economic barriers to antibiotic development and contribute to public health.

A diverse, vibrant antibiotic pipeline is a vital part of the global response to the crisis of antibacterial resistance [1]. Unfortunately, the scientific challenge of antibiotic discovery and development combines with the poor economics of antibiotics to create a strong disincentive for developers [2]. The multiple approaches being taken to address these issues include innovative public–private research partnerships [3], augmented regulatory pathways [4–6], innovative reimbursement models [1, 7], and an increase in government investment.

The availability of high-quality, disease-specific clinical trial networks would also facilitate a reliable stream of new anti-infective agents. The idea is similar to the use of networks in other areas. For example, cancer-focused networks have been able to study even very rare tumors [8]. While this example illustrates the efficiencies and discoveries made possible by such networks, networks for bacterial infections face distinctive issues.

## Sites Do Not Wish to Be Centers of Excellence for Either Nosocomial Infection or Highly Resistant Bacteria

For noninfectious diseases such as cancer or diabetes, centers of excellence usually are the cornerstones of complex trials. Potential study participants are referred (and indeed may actively seek to be referred) to such centers due to the availability of novel therapies.

However, the very nature of infectious diseases works against network creation. First, the pace of bacterial infection makes referral difficult, as delays in initiation of therapy of even a few hours can be the difference between life and death. Second, high local rates of nosocomial infections such as hospital-associated pneumonia suggest poor local infection prevention practices. Third, being a Center of Excellence for resistant bacteria because of the high rate of resistance in that locale (or, worse, that institution) is unattractive. Institutions experiencing outbreaks of resistant bacteria expend significant energy to rapidly terminate such outbreaks [9]. As a consequence, an antibiotic-focused network can usually only enroll patients presenting at a preexisting study site.

## New Agent Registration Relies on Use of Noninferiority Trial Designs

As curative therapy is expected, enrollment into antibiotic trials is ethically dubious if the infecting pathogen is resistant to the comparator. As a consequence, new development of new agents relies heavily on noninferiority design trials. In such trials, the spectrum of action of the comparator agent must be carefully considered.

Broadly, bacteria can be considered as being in 1 of 3 resistance categories: usual drug resistance (UDR), multidrug resistance (MDR), and extensive drug resistance (XDR). These categories are a continuum and reflect the ease of selection of active control therapy: UDR isolate infections are readily treated with standard therapies, whereas MDR and XDR infections require difficult and less standardized regimens.

Even though currently available therapies for MDR and XDR pathogens are in some cases of poor quality, practical considerations make it impossible to routinely design antibacterial trials with an expectation of demonstrating superiority over the comparator [4]. First, our global goal is for XDR/MDR pathogens to be rare. Second, the test agent is unlikely to demonstrate superiority over an active comparator when it is fully and properly dosed [10].

Therefore, and although making use of trial networks to study XDR/MDR pathogens is being pursued, experience to date suggests that programs focused on XDR/MDR pathogens run slowly, are costly, and have struggled to complete. Thus XDR/MDR-focused trial networks may need a different design from those studying purely UDR pathogens.

## SUSTAINABLE CLINICAL TRIAL NETWORKS FOR ANTIBACTERIAL AGENTS

As a consequence of these constraints, modern development programs are usually centered on noninferiority comparisons of the candidate drug against an effective comparator in the setting of UDR pathogens [4]. Based on this concept, we propose creation of trial networks focused on 1 of the 5 well-characterized serious infections with predictable mortality and morbidity for which high-quality study designs are now available: complicated urinary tract infection, complicated intra-abdominal infection, hospital-associated or ventilator-associated bacterial pneumonia, community-acquired bacterial pneumonia, or acute bacterial skin or skin structure infection [6, 11–16].

**Figure 1.**
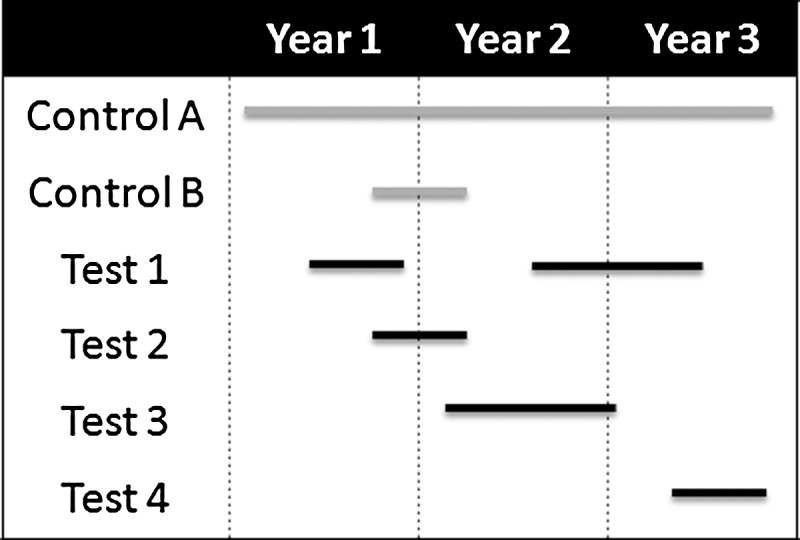
Shown are 3 years in the life of a network. To start the network, a target infection is chosen (eg, complicated intra-abdominal infection), a standard protocol is prepared, and a widely registered excellent standard comparator (control A) is selected. After a brief run-in period, test drugs are flexibly added and removed from the network. A constant control arm (control A) is envisioned, but alternative controls can also be used as needed to address issues such as blinding (control B paired with test 2). Multiple agents can be in the network simultaneously. It is also assumed that data on control agents can be shared across drugs (see text).

In such networks (Figure 1), consecutive patients with the selected infection type due to UDR pathogens could be continuously enrolled and randomized. As the standard 5 types of infection occur regularly, trial enrollment would proceed at a predictable pace. Standard of care comparator drug(s) with sufficiently broad activity and registration to permit use in most global territories would always be part of the network, and investigational drugs would flexibly rotate in and out of the randomization process.

Such a network would efficiently deliver the comparative data needed for standard phase 2 and phase 3 noninferiority studies. Product developers would still need to independently implement phase 1 studies, as well as any desired trials in infections due to MDR/XDR pathogens.

## BENEFITS FROM SUCH A NETWORK

### As Sites Gain Experience, Consistent Case Quality and Sharing of Control Data Lead to a Reduction in the Required Number of Patients

As sites accrue experience, typical trial start-up training issues will be resolved, case management processes will stabilize, and case quality and evaluability rates should reach high levels. Furthermore, if the control arm is found to be stable over time, it should be possible to share control subjects.

Such efficiencies have significant implications. Consider the impact of sharing controls across 3 drugs requiring 3 phase 2–size trials (100 subjects each on test and control) and 3 phase 3–size trials (350 subjects each on test and control) for a single indication. Independently, these trials require 2700 subjects (1350 subjects each on test and control). By sharing controls such that only 25 of every 100 enrolled subjects are randomized to control, only 1800 subjects (1350 on test, 450 on control) are needed; each drug being developed thus experiences a savings of 33%. A network enrolling approximately 500 subjects per year could deliver these data in approximately 4 years.

As the network builds a database of control-treated patients, further savings emerge. For example, pairing data on 300 test-treated patients with data on 400 control-treated patients (100 randomized in parallel, 300 from prior work) gives the same statistical power as for 350 test-treated patients paired with 350 control-treated patients. In effect, a mature network could use this strategy to generate data equivalent to that of a stand-alone 700-patient trial in the time required to enroll 400 patients, thus yielding a total time and cost reduction of 43% for that and future trials using the same comparator and protocol.

### Use of a Common High-Quality Control Addresses Concerns Regarding Biocreep

“Biocreep” is the risk that sequential noninferiority comparisons of agents (A vs B, then B vs C, then C vs D) could lead to a situation in which each comparison found the 2 agents to be noninferior but, in fact, slight reductions in efficacy are present such that D is actually inferior to A [17]. The use of a consistent, gold-standard comparator in a given network would alleviate this concern.

### Efficient Operations Speed Market Entry

Based on the authors' experience, it typically takes 3–6 months before a site completes contract negotiations, ethical approval, and other startup processes. Within an operational network, on the other hand, contracts would already be negotiated, data collection systems in place, staff trained, and the trial recruiting at the time a new drug is added. Even if only 2 months are saved during initiation for each of phase 2 and phase 3, the combined savings of 4 months is the same as the 4-month acceleration from priority review in the United States. Using standard estimates [2] and a discount rate of 12%, this increases the discounted value of future sales by 5.5% for a drug 5 years from market (eg, an additional net present value [NPV] of $55 million on a drug with a total lifetime future revenue NPV of $1 billion).

### Reduced Risk of Product Failure Due to Trial Conduct Issues

Trial conduct issues have led to product failures. Because the network would over time create a well-trained and experienced group of investigators, the risk of trial conduct issues should be minimized.

### The Network as a Springboard for Other Studies

The existence of a network could also be used as a springboard for other work. Diagnostic devices, for example, could be tested with little additional program overhead. Small companion protocols (eg, an open-label study of a test agent) could be efficiently implemented in parallel.

## CONCLUSIONS

We believe that clinical trial networks could reduce clinical trial sizes by as much as 43%, improve trial quality, and deliver time savings at least equivalent to priority review in the United States. The network would make data generation and review predictable for both regulators and developers, reduce economic barriers to entry, and provide a springboard for related studies. It should also increase our understanding of these drugs and the clinical trial process in a way that is important both for human health and future drug development.
